# Dental pulp stem cells used to deliver the anticancer drug paclitaxel

**DOI:** 10.1186/s13287-018-0831-3

**Published:** 2018-04-12

**Authors:** Hamideh Salehi, Siham Al-Arag, Elodie Middendorp, Csilla Gergely, Frederic Cuisinier, Valerie Orti

**Affiliations:** 1 LBN, University of Montpellier, Montpellier, France; 2 L2C, University of Montpellier, CNRS, Montpellier, France

**Keywords:** Raman spectroscopy, Dental pulp stem cells, Cancer, Paclitaxel, Apoptosis

## Abstract

**Background:**

Understanding stem cell behavior as a delivery tool in cancer therapy is essential for evaluating their future clinical potential. Previous in-vivo studies proved the use of mesenchymal stem cells (MSCs) for local delivery of the commonest anticancer drug, paclitaxel (PTX). Dental pulp is a relatively abundant noninvasive source of MSCs. We assess dental pulp stem cells (DPSCs), for the first time, as anticancer drug carriers. Confocal Raman microscopy is a unique tool to trace drug and cell viability without labeling.

**Methods:**

Drug uptake and cell apoptosis are identified through confocal Raman microscope. We traced translocation of cytochrome c enzyme from the mitochondria, as a biomarker for apoptosis, after testing both cancer and stem cells. The viability of stem cells was checked by means of confocal Raman microscope and by cytotoxicity assays.

**Results:**

In this study, we prove that DPSCs can be loaded in vitro with the anticancerous drug without affecting their viability, which is later released in the culture medium of breast cancer cells (MCF-7 cells) in a time-dependent fashion. The induced cytotoxic damage in MCF-7 cells was observed consequently after PTX release by DPSCs. Additionally, quantitative Raman images of intracellular drug uptake in DPSCs and MCF-7 cells were obtained. Cytotoxic assays prove the DPSCs to be more resistant to PTX as compared to bone marrow-derived MSCs, provided similar conditions.

**Conclusions:**

Applications of dental stem cells for targeted treatment of cancer could be a revolution to reduce morbidity due to chemotherapy, and to increase the efficacy of systemic cancer treatment.

## Background

Cancer is a complex heterogeneous disease; the survival of cancer patients is still poor with high mortality and morbidity rates despite recent therapeutic advances. The morbidity also results from serious side effects due to the use of nonspecific anticancerous drugs. Furthermore, in many cases, the poor survival of patients greatly relates to the inability to deliver drugs to the metastatic sites, away from the main tumor mass [1]. It is, therefore, crucial to develop new delivery strategies for chemotherapeutic drugs in clinical use.

Paclitaxel (PTX) is one of the most effective broad-spectrum anticancer drugs indicated for solid tumor malignancies, including lung, gastric, ovarian, and metastatic breast cancer [2]. PTX is a microtubule-stabilizing agent as it is believed to bind to the β-tubulin unit of the microtubules inducing mitotic arrest [3–6]. PTX toxicity includes bone marrow suppression, alopecia (100% of patients), and hypersensitivity reactions. It can also cause neurotoxicity, myalgia, and other side effects [7, 8]. The key goal of cancer chemotherapy consists of selectively localizing the drug’s effect toward the tumor in order to reduce its collateral toxicity [9–11]. Over the past decades, many tumor-selective approaches have been investigated, as antibodies, peptides for targeting tumor antigens, nanoparticles, or cellular therapy [9, 10]. Stem cells that could be expanded and modified ex vivo, and transplanted in vivo, encourage attempts to treat complex lethal diseases like cancer [12].

Mesenchymal stromal cells (MSCs) have recently gained great interest as an anticancer tool. Apart from their immunomodulatory roles, anti-inflammatory effects, secretion of bioactive molecules, and multilineage differentiating capability under appropriate conditions, their significant homing ability toward tumor and metastasis sites makes MSCs a new alternative to deliver anti-tumor agents [10, 11, 13–16]. Administration of MSCs has been associated with decreased tumor growth when injected systemically or directly in contact with tumors, suggesting their systemic effect and inhibition of tumor proliferation [16, 17]. Moreover, independently of their controversial role in inhibiting or promoting cancer growth, mesenchymal stem cells can be used as a “Trojan horse” to vehicle and deliver conventional anti-tumor agents into the cancer cells because of their ability to migrate, localize, and survive in cancer tissue, and their resistance to the chemotherapeutic drugs [15, 17–20]. When primed with PTX, human MSCs derived from the bone marrow acquire strong anti-tumor activity through their capacity to uptake, deliver, and subsequently release the internalized drug into the tumor microenvironment, thus impairing tumor growth [9, 10, 17, 21–23]. These cells have shown sensitivity to the antiproliferative activity of PTX but were strongly resistant to the drug’s cytotoxic effects even at high concentrations (> 10 μg/ml PTX) [10, 19].

In addition to the bone marrow MSCs, adipose tissue-derived mesenchymal stem cells (ADSCs) and amniotic mesenchymal stem cells (AMSCs) have been investigated [24, 25]. The potential use of ADSCs in cancer patients, especially those undergoing invasive surgery, could be affected by the disease and/or the treatment followed. Cancer patients lose their fatty tissue, which renders the autologous use of ADSCs unfeasible in this case. For AMSCs from a fetal or maternal origin, some issues have been proposed toward the isolation from different origins which may differentially express some functions not typical for MSCs, in addition to the complicated manipulation of these cells for clinical use. Allogenic or autologous stem cell transplantation may be proposed for therapy. For this reason, allogenous in-vitro expanded MSCs do not seem a promising option since xenogeneic antigens might trigger undesirable immune responses. Therefore, most of the completed clinical trials are based on autologous treatments [26].

Dental pulp is an interesting source of mesenchymal stem cells, due to the large abundance of cells from one tooth and the noninvasive isolation methods compared to other adult tissue sources [12]. Pulp tissue from human third molars, exfoliated deciduous or supernumerary teeth, represent an easy source for harvesting MSCs. The properties of dental pulp stem cells (DPSCs) distinguish them as one of the most accessible cell sources for cell-based therapy [12, 13].

Understanding the mechanism and behavior of MSCs and tracking them to check their efficacy in cancer treatment are essential for assessing their future clinical potential. Confocal Raman microscopy is the method able to track living cells [5, 6, 27–29] without a need for labeling. The laser beam being focused down to a small spot on the specimen leads to high spatial resolution and unique compositional sensitivity. This method allows cells’ analysis according to different vibrational spectra owing to differences in the biochemical composition of the molecules. The drug’s specific Raman signature enables its precise detection in the cell [5, 6, 27, 28]. Here we used MSCs of the dental pulp to assess for the first time whether they can be a promising delivery vehicle for PTX. We evaluate also by confocal Raman microscopy and cytotoxic assays the effect of PTX on the dental pulp and bone-marrow-derived mesenchymal stem cells (DPSCs and BM-MSCs), and the cytotoxic damage induced by using the in-vitro conditioned medium released from drug-loaded DPSCs to MCF-7 breast cancer cells.

## Methods

### Human dental pulp stem cells: culture and characterization

Human wisdom teeth extracted for orthodontic reasons were recovered from healthy patients (15–18 years old). Written informed consent was obtained from the parents of the patients. This protocol was approved by the local ethical committee (Comité de Protection des Personnes, Montpellier Hospital, France). Tooth surfaces were cleaned using 2% chlorhexidine and cut around the cementum–enamel junction using sterilized discs. Teeth were then broken into two pieces to reveal the pulp chamber. The pulp tissue was gently separated from the crown and root, and then digested in a solution of 3 mg/ml collagenase type I and 4 mg/ml dispase for 1 h at 37 °C. The solution was then filtered through 70-μm Falcon strainers and added to αMEM supplemented with 10% fetal bovine serum (FBS), 100 U/ml penicillin, 100 μg/ml streptomycin with the addition of 1 ng/ml basic fibroblast growth factor (bFGF) and placed in 75-ml flasks. Cells were incubated for 1 week at 37 °C with 5% CO_2_. Nonadherent cells were removed by a change of medium 24 h after cell seeding. Cells were cultivated for 24 h on polished calcium fluoride (CaF_2_) substrates (Crystran Ltd, Dorset, UK). The cultured cells were washed three times with phosphate buffered saline (PBS) to remove the culture medium, and then fixed with 2% paraformaldehyde for 15 min and rewashed with PBS before Raman imaging.

After 1 week, subconfluent cells were collected and analyzed for minimal criteria to define human mesenchymal stem cells, such as adherence to plastic, expression of cell surface antigens, and ability to differentiate into osteoblasts, adipocytes, and chondroblasts in vitro [30]. The antigen profiles of cultured DPSCs were analyzed by detecting the expression of the cell surface markers CD90, CD146, CD117, and CD45 using flow cytometry [31, 32]. CD90 is a widely accepted marker for mesenchymal stem cells, CD146 is a marker expressed in perivascular mesenchymal stem cells, CD117 is the receptor of stem cell factor, and CD45 is a marker of hematopoietic cells, mainly myeloid progenitors. The latter has been used to demonstrate the absence of contamination by CD45^+^ hematopoietic progenitors. Cells were controlled for pluripotency with in-vitro osteogenic, adipogenic, and chondrogenic differentiation essays following a previously described protocol [33].

### Human bone marrow mesenchymal stem cells

Mesenchymal stem cells expanded from human bone marrow stem cells, at their second passage, were obtained from the Institute for Regenerative Medicine and Biotherapy (IRMB, Montpellier, France). These were cultured in αMEM supplemented with 10% FBS, 100 U/ml penicillin, 100 μg/ml streptomycin, with the addition of 1 ng/ml bFGF, and were placed in 75-ml flasks. The cells were maintained at a temperature of 37 °C in humidified, concentrated CO_2_ (5%) atmosphere. All experiments were done between passages 2 and 8.

### MCF-7 cell culture

MCF-7 cells, derived from a metastatic breast cancer patient in 1970, were the first cancer cell line capable of living longer than a few months and became a standard model in cancer research laboratories [34]. MCF-7 cells were grown in 75-cm^2^ culture flasks (VWR, Strasbourg, France) in a medium containing 7 ml Dulbecco’s Modified Eagle’s Medium (DMEM) (Thermo Fisher, Strasbourg, France), 20% FBS and 1% antibiotics (streptomycin 100 μg/ml, penicillin 100 U/ml) at 37 °C and 5% CO_2_. Accordingly, to transfer the conditioned medium from stem cells to cancer cells, the medium was changed gradually in such a way that after 2 weeks MCF-7 cells were cultivated in αMEM. Cells were seeded onto polished calcium fluoride (CaF_2_) substrates (Crystran Ltd, Dorset, UK) for Raman imaging and after 24 h the cells adhered well on the CaF_2_ substrate. MCF-7 cells were first incubated in a solution of conditioned medium released by the DPSCs, and then rinsed with PBS before being transferred under the confocal Raman microscope.

### Priming with paclitaxel

In our experiments, 10 μM paclitaxel (Taxol; Teva Pharmaceutical Ind., Tel Aviv, Israel)—equivalent to the clinically used amount—was added in cell culture medium [5]. DPSCs were incubated for 12 h with 10 μM PTX. The culture medium was then removed, the cell culture was rinsed with PBS to remove noninternalized drug, and a fresh culture medium was added for 4 h. Next, MCF-7 were incubated for 3 h with the conditioned medium (CM) containing the PTX released from the DPSCs. For the cytotoxicity assays, DPSCs or BM-MSCs were incubated for 12 h with PTX at a concentration of 10 μM for viability testing.

### In-vitro cytotoxicity assay on stem cells

The effect of PTX on cell viability was evaluated by the Thiazolyl Blue Tetrazolium Bromide (MTT) assay (M-2128; Sigma-Aldrich, USA). DPSCs and bone-marrow derived MSCs were seeded on a 96-well plate (30,000 cells/well) and cultured for 12 h in the presence of PTX (concentration of 10 μM). Cell viability was calculated as the ratio between the absorbance of treated and control DPSCs. Mean and standard deviation (SD) values were generated from three replicates. Each experiment was performed at least three times. Representative results of a multiple experiments are shown.

MTT (3-(4,5-dimethylthiazol-2-yl)-2,5-diphenyl tetrazolium bromide) was dissolved in PBS at 5 mg/ml and filtered to sterilize and remove a small amount of insoluble residue present in some batches of MTT. At the times indicated in the following, a stock MTT solution (10 μl per 100 μl medium) was added to all wells, and plates were incubated at 37 °C for 4 h. After 3–4 h at 37 °C for MTT cleavage, the formazan product was solubilized by adding 0.1 ml of 0.04 N HCl isopropanol to the wells and mixing it thoroughly to dissolve the dark blue crystals [35, 36]. After less than 1 h at room temperature the plates were read on an ELX800 Micro Elisa reader (BioTek, Winooski, VT, USA), using a wavelength of 540 nm.

### Raman data acquisition and analysis

Raman spectra were collected using a Witec Confocal Raman Microscope System alpha 300R (Witec Inc., Ulm, Germany). Excitation in confocal Raman microscopy is generated by a frequency-doubled Nd:YAG laser (Newport, Irvine, CA, USA) at a wavelength of 532 nm. The incident laser beam is focused onto the sample through a 60× NIKON water immersion objective having a numerical aperture of 1.0 and a working distance of 2.8 mm (Nikon, Tokyo, Japan). The laser power after the objective is 15 mW but the power absorbed by cells in PBS is lower. The spatial resolution and depth resolution are 300 nm and 1 μm, respectively. The mixed Raman and Rayleigh scattered radiation was then passed through an edge filter to block the Rayleigh radiation from the Raman signal. The acquisition time of a single spectrum was set to 0.5 s. An area of 150 × 150 points per image was recorded leading to a total of 22,500 spectra for one image, each spectrum corresponding to a spatial unit defined as a voxel. Data acquisition was performed using Image Plus 2.08 software from Witec.

Raman data analysis is based on three methods. The first method provides integrated Raman intensities in specific spectral regions, in particular that of the C–H stretching mode providing a chemical map. Data processing is performed using Image Plus software from Witec. Using a look-up table, an image is created: bright yellow hues indicate the highest intensities and orange hues the lowest integrated intensities of the chosen region.

The second method is *K*-means cluster analysis (KMCA). *K*-means clustering partitions data into *K* mutually exclusive clusters. The *K*-mean treats each observation in the data set as an object having a location in space. It finds a partition in which objects within each cluster are as close to each other as possible, and as far from objects in other clusters as possible. KMCA was realized using Witec Project Plus (Ulm, Germany) software.

As a third analysis method, the spectral correlation matrix was calculated [37] to find the most similar spectrum to the reference spectrum of PTX. To quantify the similarity, as a “distance”, Pearson’s correlation coefficient was calculated for each pair of spectra, given by the following formula:

$$ r=\frac{\sum_{i=1}^N\left(\left({x}_i-X\right)\left({y}_i-Y\right)\right)}{\sqrt{\sum_{i=1}^N{\left({x}_i-X\right)}^2{\sum}_{i=1}^N{\left({y}_i-Y\right)}^2}}, $$

where *N* is the number of points within the spectrum, *x*_*i*_ and *y*_*i*_ are the individual points, and *X* and *Y* are the mean value of each spectrum. The value of *r* can vary between −1 and 1, and thus it can be expressed as a percentage ranging from −100% (no correlation) to 100% (the perfect match). From these values, a pseudo-color map can be constructed, reflecting the quantified similarities. All correlation calculations were performed with a homemade code written in MatLab (Math Works, Inc., Natick, MA, USA).

### Statistical analysis

Data are expressed as means, and when required the differences between mean values were analyzed by one-way ANOVA test performed by the Sigmaplot program (Systat software, San Jose, CA, USA). *p* < 0.05 was considered statistically significant.

## Results

### Cell viability results on dental pulp stem cells, bone marrow stem cells and breast cancer cells

Cell viability of dental pulp and bone marrow-derived stem cells was evaluated by MTT assay. MCF-7 cells were also tested as positive control. Optical densities at 540 nm were determined for all types of cells, treated and untreated with PTX, to compare their viability under the same conditions. The results show a higher viability for DPSCs as compared to those of BM-MSCs and MCF-7 cells, and a significant difference is found in their behavior after treatment with PTX.

For each cell type, we calculated the cell viability percentage as the ratio of the optical density of the test sample to the optical density of solvent control by the following formula:

$$ \mathrm{Cell}\ \mathrm{viability}=\frac{\mathrm{Optical}\ \mathrm{density}\ \mathrm{of}\ \mathrm{test}\ \mathrm{sample}\ \left(\mathrm{optical}\ \mathrm{density}\ \mathrm{of}\ \mathrm{test}-\mathrm{optical}\ \mathrm{density}\ \mathrm{of}\ \mathrm{blank}\right)}{\mathrm{Optical}\ \mathrm{density}\ \mathrm{of}\ \mathrm{solvent}\ \mathrm{control}\ \left(\mathrm{optical}\ \mathrm{density}\ \mathrm{of}\ \mathrm{control}-\mathrm{optical}\ \mathrm{density}\ \mathrm{of}\ \mathrm{blank}\right)}\times 100. $$

**Fig. 1:**
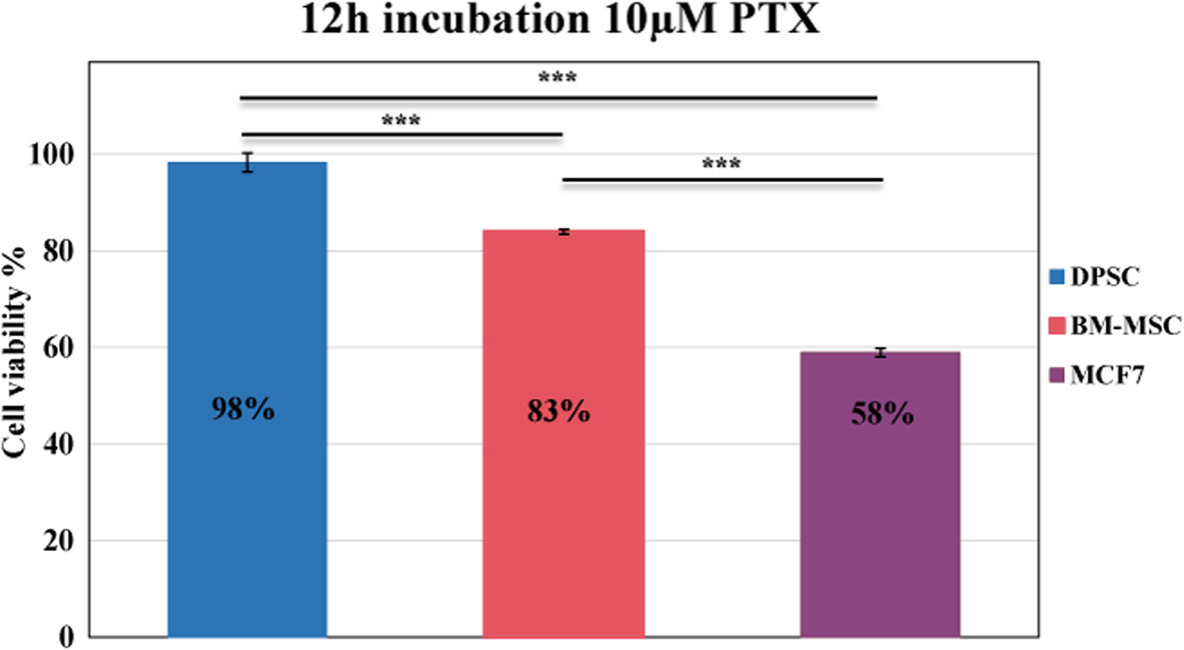
Viability assay results on control and drug-treated cells. Dental pulp and bone marrow mesenchymal stem cells incubated for 12 h with and without paclitaxel at 10 μM concentration. MCF-7 cells tested as positive control. Comparing treated DPSCs and BM-MSCs, viability measurements of test samples showed significant difference (***p < 0.001). Histogram reports mean cellular viability (%) measurement ± SD of three independent experiments. PTX paclitaxel, DPSC dental pulp stem cell, BM-MSC bone marrow-derived mesenchymal stem cell, MCF-7 Michigan Cancer Foundation-7

According to this formula, cell viability of DPSCs was found to be 98% while that of BM-MSCs was 83%, compared to 58% for the MCF-7 cells, as shown in Fig. 1.

### Raman imaging results

**Fig. 2:**
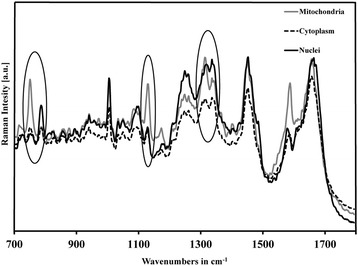
Predominant bands in Raman spectra of mitochondria (gray line), cytoplasm (dashed line), and nuclei (solid line) in cells. These peaks are used to distinguish different cell constituents

Although the spectral contrast between cellular components is relatively small, as they are very close in terms of Raman vibrations, still it is possible to reveal very small chemical differences between the various constituents of the cell. For a biological sample, the complex constituents (e.g., DNA, proteins, and lipids) in a cell generate a molecular fingerprint in the Raman spectra. Raman spectral maps of individual cells [38–40] and localization of intracellular nanoparticles [41–43] have been achieved. The average spectra of mitochondria, cytoplasm, and nuclei, calculated by KMCA, are shown in Fig. 2: the spectral peak at 750 cm^−1^ corresponds to the symmetric breathing of tryptophan (protein assignment), at 780 cm^−1^ is assigned to the (O–P–O) stretching DNA, at 1128 cm^−1^ is the ν(C–C) skeletal acyl backbone in lipid, at 1312 cm^−1^ is the (CH_3_CH_2_) twisting mode of lipid, and at 1335 cm^−1^ is adenine, guanine (ring breathing modes in the DNA bases), as reported in the literature [44]. The relative ratio between these peaks would help to distinguish between the different cell organelles.

**Fig. 3:**
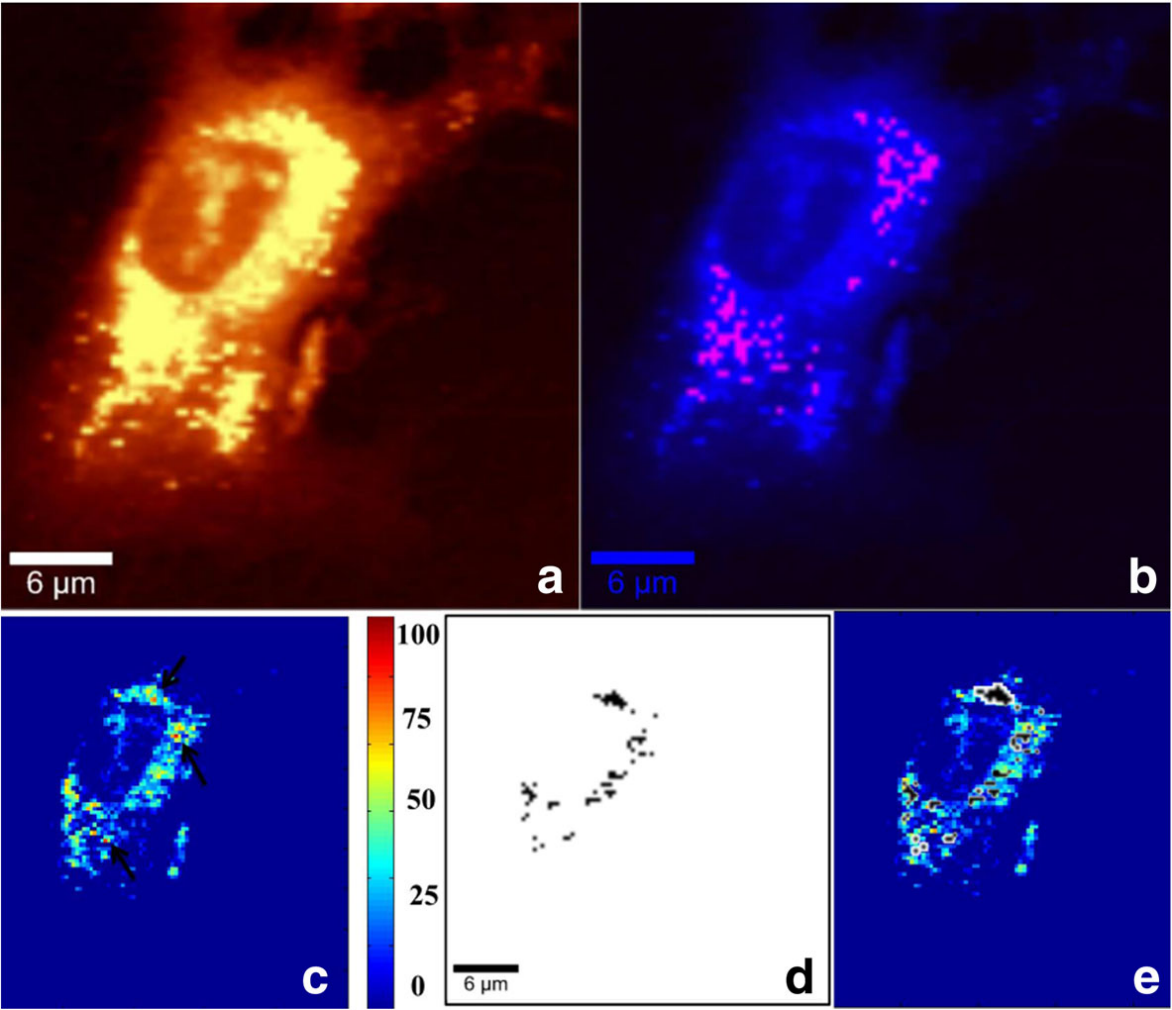
PTX uptake by DPSCs (Step 1). Incubation for 12 h with 10 μM PTX PBS solution, 60× water immersion objective. **a** Integrated Raman intensities in 2800–3000 cm^−1^ region of cells corresponding to C–H mode. **b** KMCA image to detect intracellular PTX (pink spots). **c** Pearson’s correlation map between whole cell Raman spectra and reduced cytochrome c Raman spectrum. Highest correlation obtained for red spots (indicated by black arrows). Region with no correlation to cytochrome c (navy blue) corresponds to fresh PBS buffer. **d** Mitochondria cluster (black) obtained from KMCA. **e** Mitochondria cluster overlapped in correlation map of cytochrome c; all positions corresponding to cytochrome c completely covered by the mitochondria cluster that indicates localization of cytochrome c within mitochondria

For a better follow-up, we summarize here the main steps of the experiments we have performed:

**Step 1:** Paclitaxel is added to DPSCs (12-h incubation with 10 μM paclitaxel). DPSC uptake of the anticancer drug is monitored (Fig. 3).

**Fig. 4:**
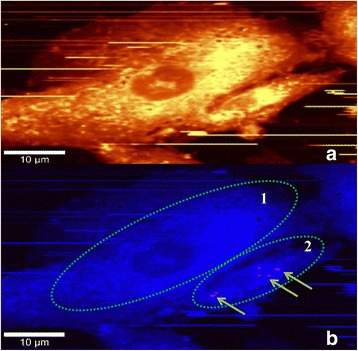
Two DPSCs after 4-h release of PTX in a PBS solution imaged in confocal Raman microscopy (Step 2). **a** Integrated Raman intensities in 2800–3000 cm^−1^ region of cells corresponding to C–H mode. **b** Raman reconstruction of **a**, using KMCA to detect intracellular PTX (pink spots). Cell 1 shows no intracellular drug while cell 2 contains PTX (indicated by arrows)

**Fig. 5:**
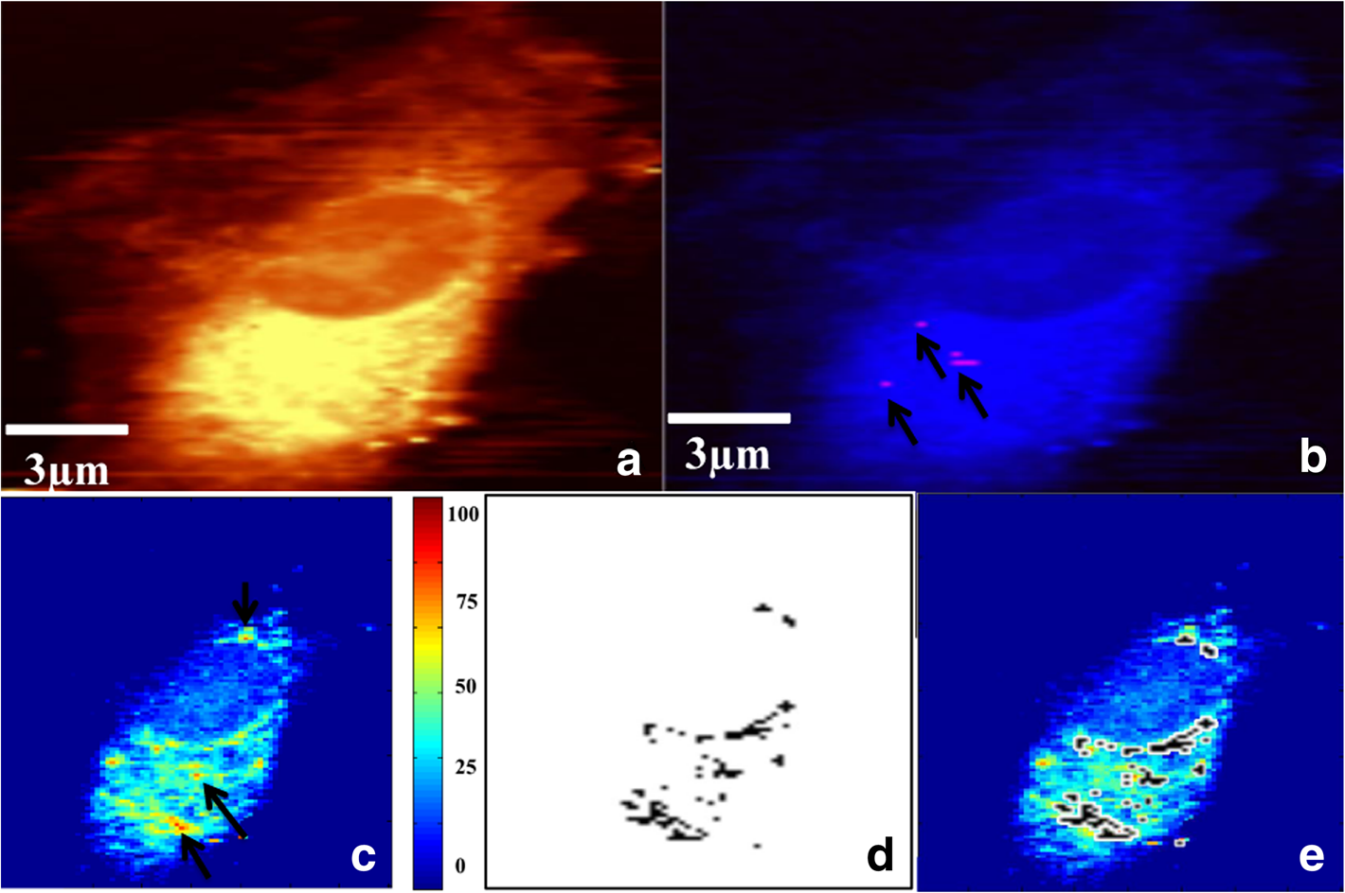
Viability of DPSCs after releasing PTX. **a** Integrated Raman intensities in 2800–3000 cm^–1^ region of cells. **b** Raman reconstruction of **a**, using KMCA to detect intracellular PTX (pink spots). **c** Correlation map and correlation coefficient between whole cell image and image of cytochrome c. Best correlation obtained for red pixels (indicated by black arrows): region with highest correlation between reference spectrum of cytochrome c and their spectra in cells. Region with no correlation to cytochrome c is due to PBS buffer (blue region). **d** Mitochondrial cluster (black) obtained from KMCA. **e** Mitochondrial cluster overlapped with correlation map of cytochrome c. All positions corresponding to cytochrome c are covered, indicating localization of cytochrome c inside mitochondria

**Step 2:** DPSCs release paclitaxel into fresh culture medium. After the release of paclitaxel, this medium is called conditioned medium (Figs. 4 and 5).

**Fig. 6:**
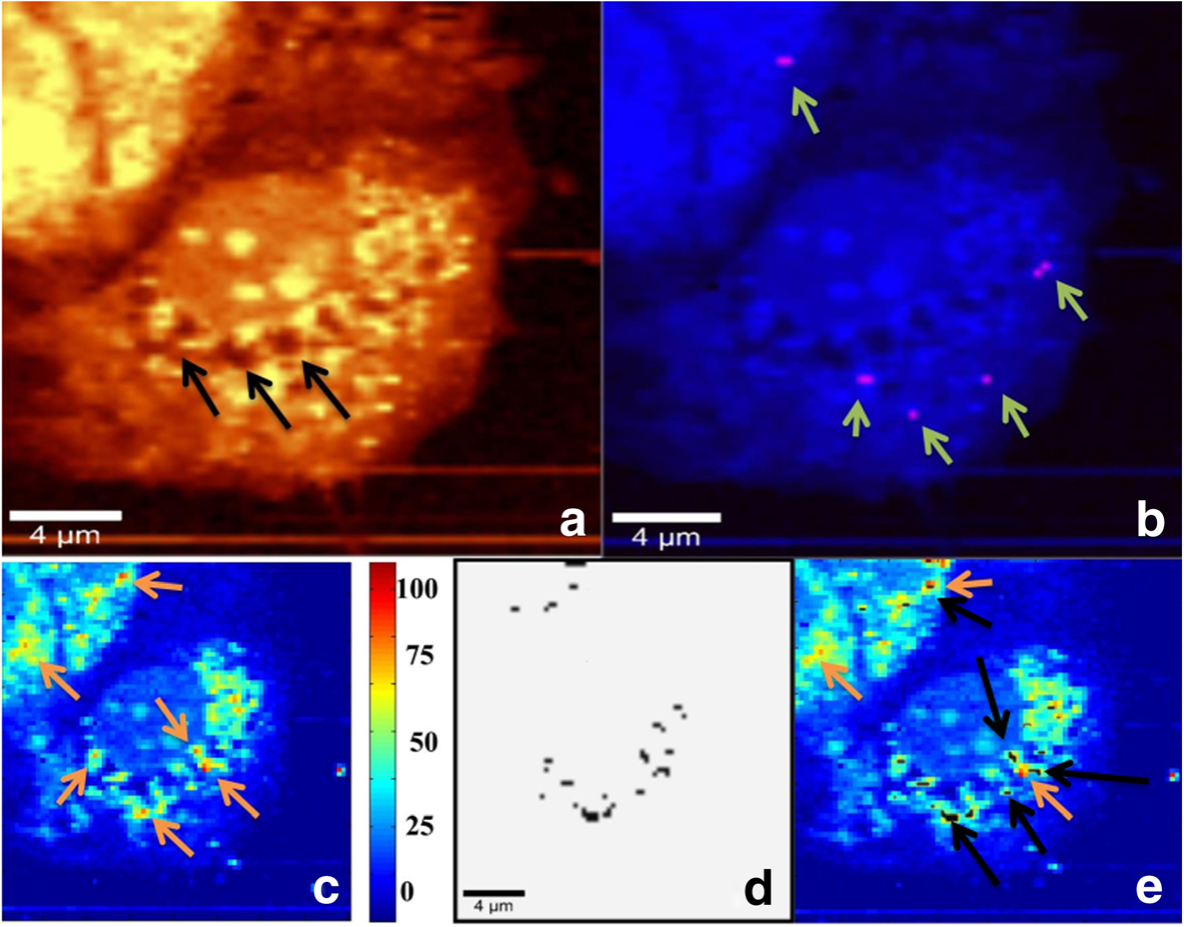
MCF-7 cells after PTX uptake from conditioned medium released by DPSCs **(**Step 3). **a** Integrated Raman intensities in 2800–3000 cm^-1^ region of cells (black arrows indicates cell's distruction). **b** Raman reconstruction of a, using KMCA to detect intracellular PTX (pink spots, indicated by green arrows). **c** Correlation map and correlation coefficient between whole cell image spectra (treated cells for 3 h with conditioned medium released by DPSCs) and image of cytochrome c. Best correlation obtained for red pixels (indicated by orange arrows): region with highest correlation between reference spectrum of cytochrome c and their spectra in cells. Region with no correlation to cytochrome c is due to PBS buffer (blue region). **d** Mitochondria cluster (black) obtained from KMCA. **e** Mitochondria cluster overlaps with correlation map of cytochrome c. Orange arrows show pixels of cytochrome c not covered by mitochondrial cluster (indicated by black arrows), indicating release of cytochrome c from mitochondria during apoptosis

**Fig. 7:**
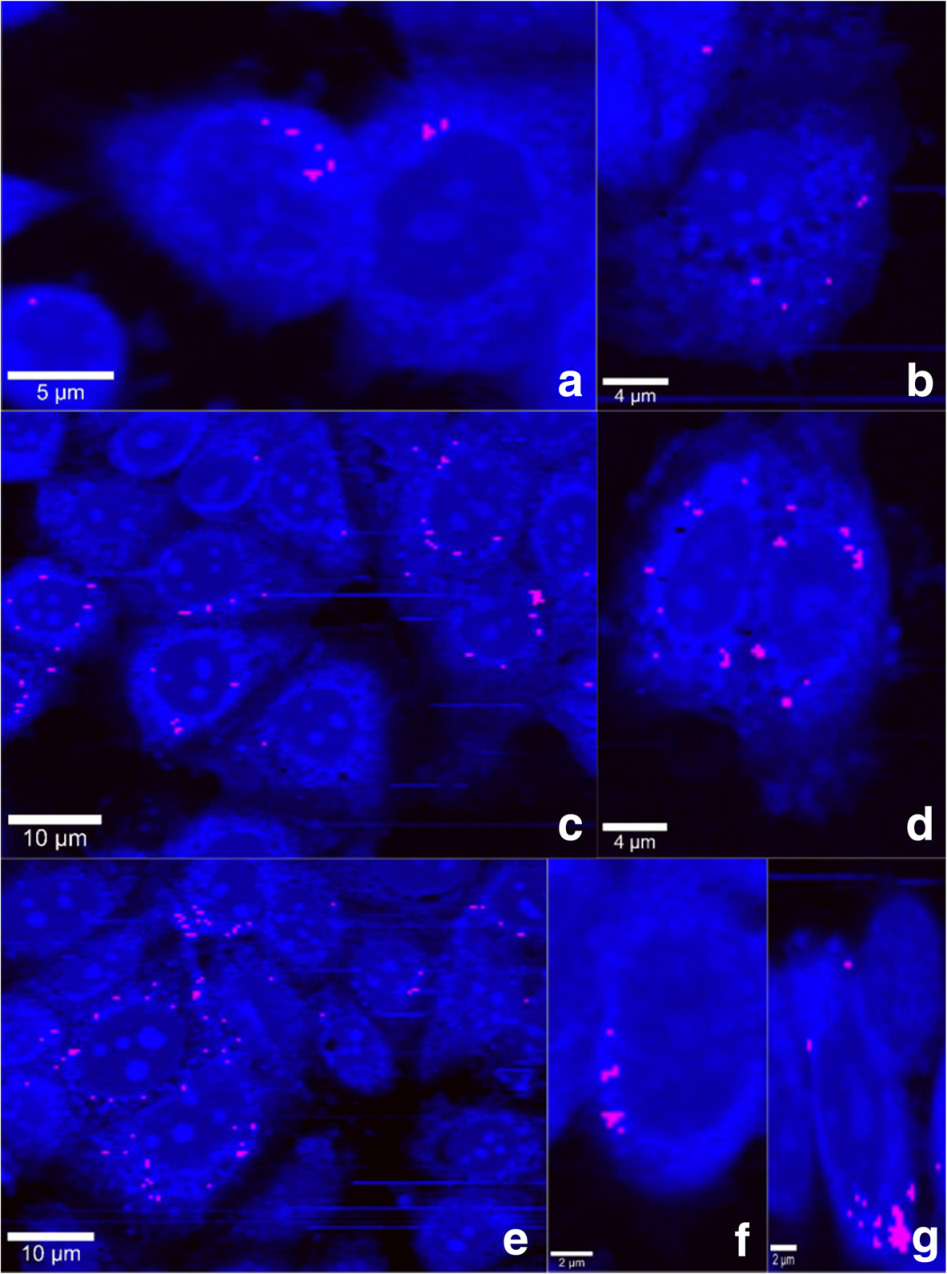
MCF-7 cells in buffer solution (Step 3). **a**–**g** Raman images of MCF-7 cells loaded with PTX (pink pixels), obtained via KMCA

**Step 3:** MCF-7 is incubated in conditioned medium containing paclitaxel (Figs. 6 and 7).

**Step 1:** First, the ability of stem cells to uptake a high concentration of the anticancer drug is verified. After incubation for 12 h with 10 μM paclitaxel, DPSCs are rinsed five times with culture medium and three times with PBS. Figure 3a shows a DPSC Raman image after step 1. It refers to the total integrated Raman intensities in specific spectrum regions (2800–3000 cm^−1^) corresponding to the C–H mode. By choosing the specific CH mode spectral region in the sum filter tool, we could create an image based on integrated Raman intensities of the C–H mode. In Fig. 3a, bright yellow hues indicate high intensities of intracellular C–H stretching modes, while dark hues (no C–H) correspond to PBS. Figure 3b shows paclitaxel uptake by DPSCs. The red pixels correspond to the intracellular drug. The drug map is calculated by *K*-mean cluster analysis (KMCA), a method described in previous publications [5]. To obtain information about cellular viability, a specific Raman approach is applied based on the detection of released cytochrome c outside of the mitochondria on the apoptotic path [6]. Briefly, a cytochrome c map is constituted based on the Pearson’s correlation coefficient map obtained from the reference Raman spectrum of cytochrome c and the whole spectra of the image. Figure 3c depicts the cytochrome c map, the red pixels marked by black arrows being the positions with a high concentration of intracellular cytochrome c. If the cell is going through apoptosis, the mitochondrial cluster should not be superimposed on the cytochrome c map; that is, cytochrome c is released from the mitochondria when the cell enters through apoptosis. If cytochrome c is colocalized with the mitochondrial cluster, there is no apoptosis. Figure 3d shows the mitochondrial cluster obtained by KMCA. The superposition of images from Fig. 3c, d is presented in Fig. 3e, clearly indicating that the mitochondrial cluster overlays completely all of the cytochrome c, which is the case for nonapoptotic cells. All of the 15 analyzed DPSCs showed uptake of paclitaxel without apoptosis.

**Step 2:** DPSCs were kept in the culture medium for 4 h to release the paclitaxel. After rinsing in PBS, cells were transferred to the Raman microscope to verify whether all of the paclitaxel was released from the DPSCs. Figure 4 shows two DPSCs in PBS. Figure 4a presents the total integrated Raman intensities corresponding to the C–H mode of two cells. Paclitaxel’s higher concentration corresponds to red pixels in Fig. 4b, where the two cells are marked with ovals. Cell 2 shows intracellular paclitaxel indicated by a few pixels, while the other cell has no detectable paclitaxel.

It is of interest to check DPSC viability after their incubation in paclitaxel-containing culture medium and the release of paclitaxel (Fig. 5). As for cells of Fig. 4, some paclitaxel is still present in the cells even after 4 h. The Raman map of cytochrome c and the cluster of mitochondria are perfectly superposed (Fig. 5e). This indicates that cytochrome c is still inside mitochondria and that apoptosis has not yet started.

**Step 3:** Breast cancer cells (MCF-7 cells) were incubated for 3 h in the culture medium—namely the conditioned medium (CM)—containing the paclitaxel released by DPSCs. The same number of cells for MCF-7 cells and DPSCs was used for each set of experiments.

Figures 6b and 7 showed MCF-7 cells containing paclitaxel (pink pixels). A total of 36 cancerous cells out of 42 showed the presence of paclitaxel. In Fig. 6e, the partial superposition of the mitochondrial cluster on the cytochrome c correlation map indicates the start of apoptosis.

## Discussion

Our work addresses the quest for powerful strategies for carrying therapeutic agents straight to the targeted tumor using mesenchymal stem cells as vehicles, and consequently for local delivery of drugs at therapeutic concentrations. This has been proposed and validated by previous in-vitro studies [10, 17, 20–25, 45] and in-vivo studies [10, 21, 46] for bone marrow-derived MSCs. DPSCs as an easy noninvasive source of mesenchymal stem cells are a promising cargo for drugs. The path of paclitaxel (PTX), a classical anticancer drug, transported by DPSCs and absorbed by MCF-7 cells, was successfully monitored by means of confocal Raman microscopy. This label-free imaging method enabled detection of the cellular organelles (mitochondria), biological molecules, such as cytochrome c, and also tracing of the drug PTX within the cell [5, 6, 27]. DPSCs showed a great capacity to uptake PTX. The cytotoxic assays showed that DPSCs were found more resistant to PTX cytotoxicity compared to BM-MSCs under the same conditions.

We developed a Raman spectroscopic imaging technique to detect intracellular PTX while simultaneously verifying the viability of DPSCs by monitoring the eventual release of cytochrome c from the mitochondria as one path of cell apoptosis [5]. After PTX uptake, the viability of the relevant cell was checked and, surprisingly, despite the high concentration of drug, no apoptosis was observed in DPSCs, demonstrating their robustness and appropriateness to vehicle PTX. Previous research showed that PTX treatment does not induce apoptosis in human bone marrow MSCs [9, 10, 17, 21, 23], and no perceivable effective concentration could be determined to initiate apoptosis within those cells [19].

We observed the release of PTX by DPSCs during 4 h in the culture medium. DPSCs act as a reservoir for PTX, and the cellular concentration of drug decreases within 4 h. This reservoir after being removed to a fresh culture medium starts to release its load, the drug and specific factors near the breast cancer cells. Paclitaxel was detected in the MCF-7 cells by Raman microscopy, and apoptosis was observed. In total, 100% of the DPSCs were successfully loaded with the drug, while almost 86% of the MCF-7 cells uptake it from the conditioned medium. The transportation of drug by DPSCs might have an effect on drug bioavailability, as the apoptosis could be observed already after 3 h in conditioned medium released from DPSCs (step 3). The drug concentration in the secreted vesicles after 3-h incubation with DPSC-CM should be equal to or higher than 0.5 μM [5].

To the best of our knowledge, the current work is the first presenting a DPSC model as a paclitaxel delivery vehicle. The other novelty of our proof of concept is the use of chemical mapping of cells to visualize PTX inside the living cells without labeling but based on Raman biochemical signals. Fluorescence labeling as an alternative method is not possible for small drug particle tracking without losing its activity, mainly because the fluorescence labels are bigger than the active molecules, and thus their introduction may change the molecule’s biochemical properties.

## Conclusion

Our results point toward the auspicious use of DPSC cells for cancer therapy. These cells are indeed efficient vehicles having the ability to uptake, migrate toward the cancer, and deliver paclitaxel without undergoing apoptosis. Raman spectroscopy can be further used to reveal the effect of paclitaxel exposure upon the function and viability of stem cells. Injection of DPSCs for targeted drug delivery against cancer cells is a promising approach to avoid the side effects of systemic PTX delivery and increase the efficacy of treatment.

## Abbreviations

ADSC: Adipose tissue-derived stem cell
AMSC: Amniotic mesenchymal stem cell
BM-MSC: Bone marrow-derived mesenchymal stem cell
C–H: Carbon hydrogen mode
CM: Conditioned medium
DMEM: Dulbecco’s Modified Eagle’s Medium
DPSC: Dental pulp stem cells
FBS: Fetal bovine serum
KMCA: *K*-means cluster analysis
MCF-7: Michigan Cancer Foundation-7
MSC: Mesenchymal stem cell
MTT: Methyl thiazolyl tetrazolium
Nd:YAG: Neodymium-doped Yttrium Aluminum Garnet
PBS: Phosphate-buffered saline
PTX: Paclitaxel
SD: Standard deviation
αMEM: Alpha Modified Eagle’s Medium
